# Supplementary material for: Is maternal autonomy associated with child nutritional status? Evidence from a cross-sectional study in India
Source: PLoS One. 2022 May 11;17(5):e0268126. doi: 10.1371/journal.pone.0268126 (PMC9094570; doi:10.1371/journal.pone.0268126)
Supplement: Attachment — Submitted filename: Response to Reviewers.docx [file pone.0268126.s002.docx]

Response to Reviewers:

Thank you so much for your constructive comments and suggestions for improving our paper.

**Reviewer #1:**

Line 217-220: author mentioned that “After excluding few samples, our results still represent the original population.” in the response. But, I suggest the author provide the result in supplementary files to present the result for comparing the original dataset (45231 mother-child pairs) and sub-dataset (38625 mother-child pairs) to prove the representative.

**Response**: Our analysis is based on the state module. However, we had to exclude a few cases from that module due to missing cases in outcome variables (i.e., cases that are out of plausible limit and flagged cases which are indicated in Figure 1). Therefore, it is not possible to conduct analysis from samples of the entire state module. We believe that the exclusion of a few samples from the state module does not change the representativeness of the data.

Table 3-5: “Two model 3 and without model 5 in the table.”

**Response**: It has been fixed now.

**Reviewer #2:** (No Response)

Thank you very much for suggesting that our manuscript does not need any further revision.
